# Tetra­aqua­bis(pyridine-κ*N*)cobalt(II) bis­[4-amino-*N*-(6-chloro­pyridazin-3-yl)benzene­sulfonamidate]

**DOI:** 10.1107/S1600536809049599

**Published:** 2009-11-25

**Authors:** Nan Li, Hong-Li Zou, Xiao-Yan Song, Yan-Cheng Liu, Zhen-Feng Chen

**Affiliations:** a Key Laboratory for the Chemistry and Molecular Engineering of Medicinal Resources (Ministry Education of China), School of Chemistry & Chemical Engineering, Guangxi Normal University, Guilin 541004, People’s Republic of China

## Abstract

The structure of the title compound, [Co(C_5_H_5_N)_2_(H_2_O)_4_](C_10_H_8_ClN_4_O_2_S)_2_, consists of a discrete tetra­aqua­bis(pyridine-κ*N*)cobalt(II) cation and two 4-amino-*N*-(6-chloro­pyridazin-3-yl)benzene­sulfonamidate anions. In the cation, the Co^II^ ion sits on an inversion centre and is octa­hedrally coordinated by two pyridine N atoms and four O atoms. A two-dimensional network parallel to (010) is formed *via* inter­molecular O—H⋯O, O—H⋯N, N—H⋯N and N—H⋯O hydrogen bonds.

## Related literature

For the structure of sulfachloro­pyridazine, see: Tan *et al.* (2005 ▶). For a sulfachloro­pyridazine–metal complex, see: Fogg *et al.* (1995 ▶). For an aqua­pyridine–cobalt(II) complex, see: Clegg *et al.* (2006 ▶).
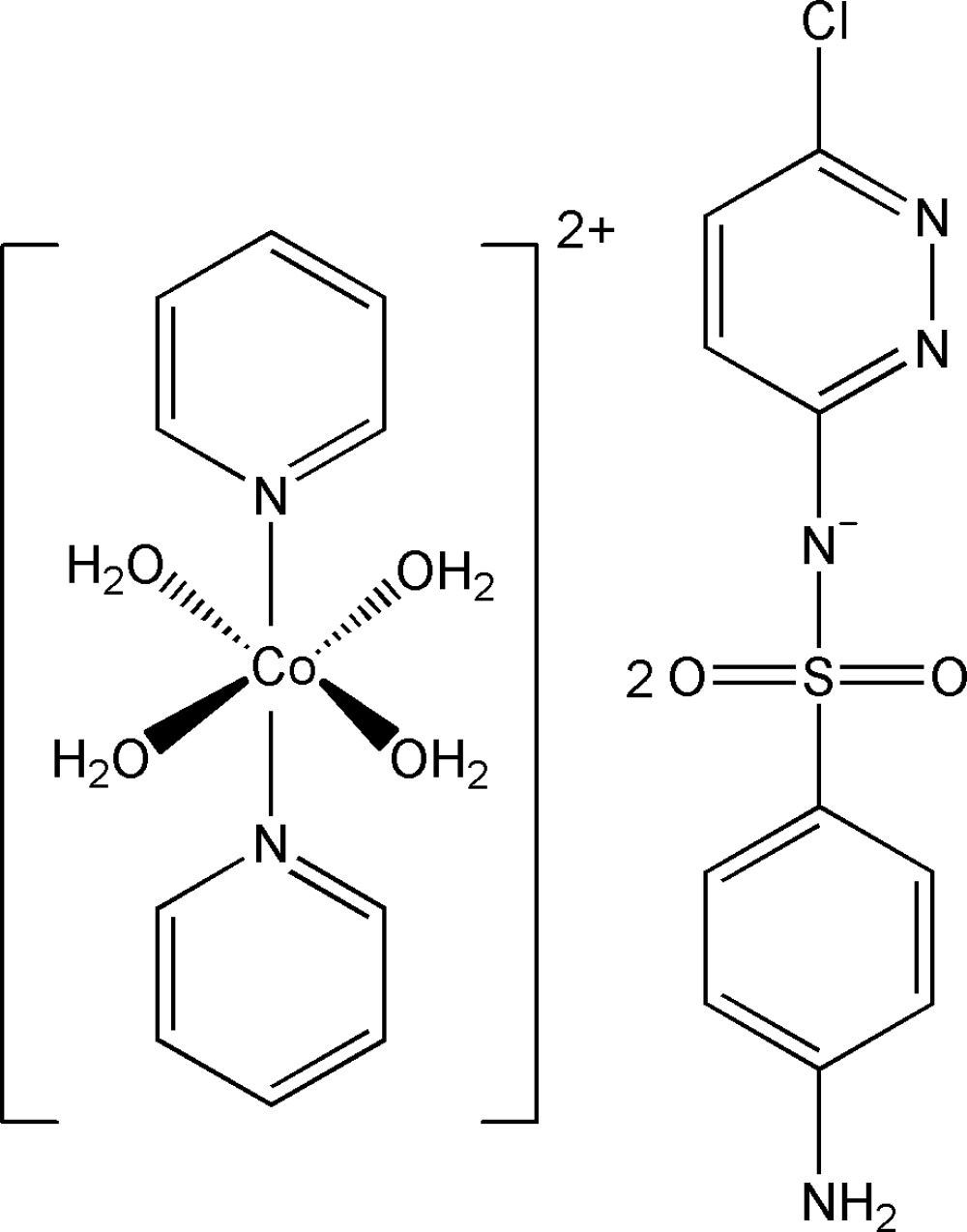


## Experimental

### 

#### Crystal data

[Co(C_5_H_5_N)_2_(H_2_O)_4_](C_10_H_8_ClN_4_O_2_S)_2_
*M* *_r_* = 856.62
Monoclinic, 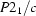

*a* = 8.5897 (12) Å
*b* = 25.807 (3) Å
*c* = 8.5338 (12) Å
β = 101.694 (3)°
*V* = 1852.5 (4) Å^3^
*Z* = 2
Mo *K*α radiation
μ = 0.78 mm^−1^
*T* = 193 K
0.21 × 0.15 × 0.12 mm

#### Data collection

Rigaku Mercury CCD diffractometer
Absorption correction: multi-scan (*REQAB*; Jacobson, 1998 ▶) *T*_min_ = 0.853, *T*_max_ = 0.912
20359 measured reflections
4226 independent reflections
3331 reflections with *I* > 2σ(*I*)
*R*_int_ = 0.050

#### Refinement

*R*[*F* ^2^ > 2σ(*F* ^2^)] = 0.054
*wR*(*F* ^2^) = 0.108
*S* = 1.14
4226 reflections
258 parameters
1 restraint
H atoms treated by a mixture of independent and constrained refinement
Δρ_max_ = 0.66 e Å^−3^
Δρ_min_ = −0.45 e Å^−3^

### 

Data collection: *CrystalClear* (Rigaku, 1999 ▶); cell refinement: *CrystalClear*; data reduction: *CrystalStructure* (Rigaku/MSC and Rigaku, 2000 ▶); program(s) used to solve structure: *SHELXS97* (Sheldrick, 2008 ▶); program(s) used to refine structure: *SHELXL97* (Sheldrick, 2008 ▶); molecular graphics: *SHELXTL* (Sheldrick, 2008 ▶); software used to prepare material for publication: *SHELXTL*.

## Supplementary Material

Crystal structure: contains datablocks I, global. DOI: 10.1107/S1600536809049599/pk2211sup1.cif

Structure factors: contains datablocks I. DOI: 10.1107/S1600536809049599/pk2211Isup2.hkl

Additional supplementary materials:  crystallographic information; 3D view; checkCIF report

## Figures and Tables

**Table 1:** Hydrogen-bond geometry (Å, °)

*D*—H⋯*A*	*D*—H	H⋯*A*	*D*⋯*A*	*D*—H⋯*A*
N3—H3*A*⋯N5^i^	0.88	2.52	3.135 (4)	127
N3—H3*B*⋯O3^ii^	0.88	2.23	3.001 (3)	147
O1—H1*A*⋯O3	0.81 (4)	2.07 (4)	2.874 (3)	174 (4)
O1—H1*B*⋯N4^iii^	0.83 (4)	2.01 (4)	2.838 (3)	178 (3)
O2—H2*A*⋯O4	0.82 (3)	1.93 (4)	2.726 (3)	165 (4)
O2—H2*B*⋯N2^iv^	0.82 (4)	1.96 (4)	2.768 (3)	170 (4)
O2—H2*B*⋯O4^iv^	0.82 (4)	2.62 (4)	3.142 (3)	123 (3)

Symmetry codes: (i) 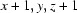; (ii) 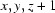; (iii) 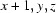; (iv) 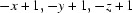.

**Fig. 1.**
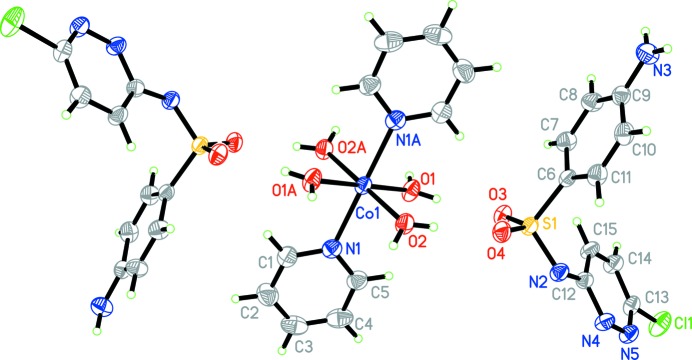
The molecular structure showing the atom-labelling scheme. Displacement ellipsoids are drawn at the 50% probability level.

**Fig. 2.**
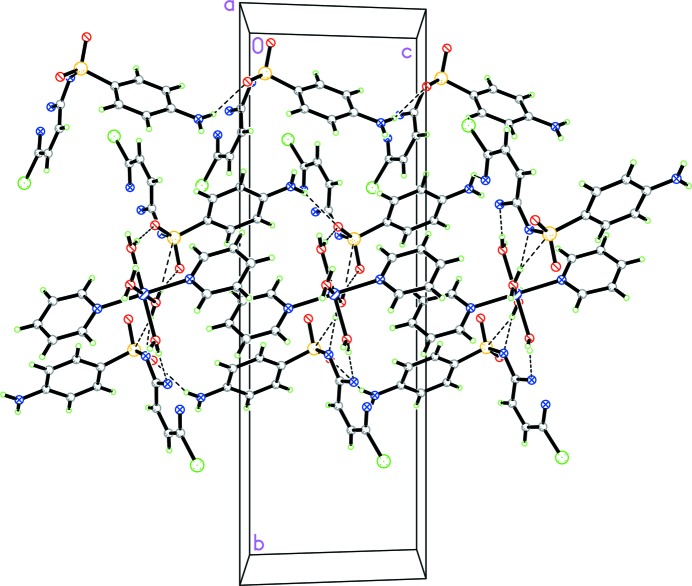
A packing diagram of title compound, viewing down the [100] direction. Hydrogen bonds are shown as dashed lines.

## supplementary crystallographic information

### Comment

Sulfachloropyridazine [4-amino-*N*-(6-chloro-3-pyridazinyl)-benzensulfonamide], is a synthetic sulfanilamide antibacterial drug whose crystal structure is known (Tan *et al.* 2005). However, as far as we are aware, no crystal structure of a metal complex containing sulfachloropyridazine has been published, although the electrochemistry of its copper(I) complex has been described by Fogg *et al.* (1995). The compound consists of a [Co(H_2_O)_4_(py)_2_]^2+^ cation and two sulfacholoropyridazine anions. Similar to tetraaquabis(pyridine-κ*N*)cobalt(II) diacetate (Clegg *et al.*, 2006), the coordination geometry for the Co^II^ cation (Fig. 1) is close to an ideal octahedron (N1—Co1—N1^#1^ 180.000 (1)°, O1—Co1—O2 92.37 (9)°, O1—Co1—O2^#1^ 87.63 (9)°, #1: -*x* + 2,-*y* + 1,-*z* + 1), with the O atoms of the coordinated water molecules occupying the equatorial positions and with the axial sites occupied by coordinated pyridine ligands. The sulfachloropyridazine is depronated at sulfamide N2 to generate anions, whose geometric parameters are comparable to sulfachloropyridazine (Tan *et al.*, 2005). A two-dimensional network is formed *via* intermolecular hydrogen bonds of type O—H···O, O—H···N and N—H···O (Table 1).

### Experimental

Samples of sulfachloropyridazine (0.2 mmol) and Co(Ac)_2_.4H_2_O (0.1 mmol) were placed in a thick-walled Pyrex tube (*ca* 20 cm long). After addition of ethanol (2.2 ml), H_2_O (0.2 ml) and pyridine (0.1 ml), the tube was frozen with liquid nitrogen, evacuated under vacuum and sealed with a torch. The tube was heated at 80°C for 3 days and then was slowly cooled down to room temperature, and orange-yellow block-shaped crystals were obtained. Yield: 80%.

### Refinement

The H atoms bonded to C atoms were positioned geometrically and refined using a riding model with *U*_iso_(H) = 1.2*U*_eq_(C) (C—H = 0.95 Å). The H atoms attached to amino N were placed in the calculated positions, with N—H distance of 0.88 Å. The *U*_iso_(H) values were constrained to be 1.2*U*_eq_ of the carrier atom for amino H atom. H atom attached to O was located in an electron-density difference map and refined isotropically.

### Crystal data

**Table:**

[Co(C_5_H_5_N)_2_(H_2_O)_4_](C_10_H_8_ClN_4_O_2_S)_2_	*F*(000) = 882
*M**_r_* = 856.62	*D*_x_ = 1.536 Mg m^−^^3^
Monoclinic, *P*2_1_/*c*	Mo *K*α radiation, λ = 0.71070 Å
Hall symbol: -P 2ybc	Cell parameters from 6394 reflections
*a* = 8.5897 (12) Å	θ = 3.1–27.5°
*b* = 25.807 (3) Å	µ = 0.78 mm^−^^1^
*c* = 8.5338 (12) Å	*T* = 193 K
β = 101.694 (3)°	Block, orange-yellow
*V* = 1852.5 (4) Å^3^	0.21 × 0.15 × 0.12 mm
*Z* = 2	

### Data collection

**Table:**

Rigaku Mercury CCD diffractometer	4226 independent reflections
Radiation source: fine-focus sealed tube	3331 reflections with *I* > 2σ(*I*)
graphite	*R*_int_ = 0.050
Detector resolution: 7.31 pixels mm^-1^	θ_max_ = 27.5°, θ_min_ = 3.2°
ω scans	*h* = −11→9
Absorption correction: multi-scan (REQAB; Jacobson, 1998)	*k* = −32→33
*T*_min_ = 0.853, *T*_max_ = 0.912	*l* = −10→11
20359 measured reflections	

### Refinement

**Table:**

Refinement on *F*^2^	Primary atom site location: structure-invariant direct methods
Least-squares matrix: full	Secondary atom site location: difference Fourier map
*R*[*F*^2^ > 2σ(*F*^2^)] = 0.054	Hydrogen site location: inferred from neighbouring sites
*wR*(*F*^2^) = 0.108	H atoms treated by a mixture of independent and constrained refinement
*S* = 1.14	*w* = 1/[σ^2^(*F*_o_^2^) + (0.0291*P*)^2^ + 1.7941*P*] where *P* = (*F*_o_^2^ + 2*F*_c_^2^)/3
4226 reflections	(Δ/σ)_max_ < 0.001
258 parameters	Δρ_max_ = 0.66 e Å^−^^3^
1 restraint	Δρ_min_ = −0.44 e Å^−^^3^

### Special details

**Table:**

Geometry. All e.s.d.'s (except the e.s.d. in the dihedral angle between two l.s. planes) are estimated using the full covariance matrix. The cell e.s.d.'s are taken into account individually in the estimation of e.s.d.'s in distances, angles and torsion angles; correlations between e.s.d.'s in cell parameters are only used when they are defined by crystal symmetry. An approximate (isotropic) treatment of cell e.s.d.'s is used for estimating e.s.d.'s involving l.s. planes.
Refinement. Refinement of *F*^2^ against ALL reflections. The weighted *R*-factor *wR* and goodness of fit *S* are based on *F*^2^, conventional *R*-factors *R* are based on *F*, with *F* set to zero for negative *F*^2^. The threshold expression of *F*^2^ > σ(*F*^2^) is used only for calculating *R*-factors(gt) *etc*. and is not relevant to the choice of reflections for refinement. *R*-factors based on *F*^2^ are statistically about twice as large as those based on *F*, and *R*- factors based on ALL data will be even larger.

### Fractional atomic coordinates and isotropic or equivalent isotropic displacement parameters (Å^2^)

**Table:**

	*x*	*y*	*z*	*U*_iso_*/*U*_eq_	
Co1	1.0000	0.5000	0.5000	0.02572 (15)	
Cl1	0.07781 (10)	0.20506 (3)	0.22874 (12)	0.0489 (2)	
S1	0.54834 (8)	0.39936 (3)	0.61366 (9)	0.02737 (18)	
O1	0.9392 (3)	0.42160 (8)	0.4313 (3)	0.0348 (5)	
O2	0.7795 (2)	0.51495 (9)	0.5400 (3)	0.0337 (5)	
O3	0.6620 (2)	0.37878 (8)	0.5233 (2)	0.0330 (5)	
O4	0.5622 (2)	0.45443 (7)	0.6462 (2)	0.0331 (5)	
N1	0.9177 (3)	0.52499 (9)	0.2536 (3)	0.0315 (6)	
N2	0.3692 (3)	0.39029 (9)	0.5297 (3)	0.0299 (6)	
N3	0.7205 (3)	0.30830 (10)	1.2615 (3)	0.0394 (6)	
H3A	0.7967	0.2850	1.2827	0.047*	
H3B	0.6729	0.3190	1.3377	0.047*	
N4	0.1620 (3)	0.34401 (9)	0.3915 (3)	0.0309 (6)	
N5	0.0921 (3)	0.30070 (9)	0.3205 (3)	0.0323 (6)	
C1	0.9858 (4)	0.56332 (13)	0.1875 (4)	0.0405 (8)	
H1C	1.0705	0.5817	0.2530	0.049*	
C2	0.9403 (4)	0.57775 (15)	0.0292 (4)	0.0479 (9)	
H2C	0.9928	0.6054	−0.0125	0.057*	
C3	0.8187 (4)	0.55177 (14)	−0.0666 (4)	0.0457 (9)	
H3	0.7851	0.5609	−0.1760	0.055*	
C4	0.7458 (4)	0.51210 (14)	−0.0018 (4)	0.0469 (9)	
H4	0.6609	0.4933	−0.0657	0.056*	
C5	0.7977 (4)	0.49995 (12)	0.1575 (4)	0.0378 (7)	
H5	0.7463	0.4726	0.2015	0.045*	
C6	0.5891 (3)	0.36813 (10)	0.8007 (3)	0.0262 (6)	
C7	0.7082 (4)	0.33130 (12)	0.8356 (4)	0.0339 (7)	
H7	0.7613	0.3199	0.7545	0.041*	
C8	0.7499 (4)	0.31125 (12)	0.9877 (4)	0.0355 (7)	
H8	0.8307	0.2856	1.0101	0.043*	
C9	0.6757 (3)	0.32790 (11)	1.1094 (3)	0.0299 (6)	
C10	0.5524 (4)	0.36397 (12)	1.0716 (4)	0.0349 (7)	
H10	0.4976	0.3750	1.1519	0.042*	
C11	0.5097 (3)	0.38357 (11)	0.9191 (4)	0.0334 (7)	
H11	0.4252	0.4079	0.8946	0.040*	
C12	0.3157 (3)	0.34323 (11)	0.4672 (3)	0.0275 (6)	
C13	0.1763 (3)	0.25836 (11)	0.3265 (4)	0.0315 (7)	
C14	0.3340 (4)	0.25379 (11)	0.4036 (4)	0.0351 (7)	
H14	0.3898	0.2219	0.4054	0.042*	
C15	0.4049 (3)	0.29672 (11)	0.4761 (4)	0.0328 (7)	
H15	0.5124	0.2957	0.5321	0.039*	
H1A	0.859 (5)	0.4089 (15)	0.451 (5)	0.060 (13)*	
H1B	1.005 (4)	0.3990 (13)	0.422 (4)	0.038 (10)*	
H2A	0.717 (4)	0.4932 (12)	0.559 (5)	0.070 (14)*	
H2B	0.734 (4)	0.5420 (15)	0.508 (5)	0.058 (12)*	

### Atomic displacement parameters (Å^2^)

**Table:**

	*U*^11^	*U*^22^	*U*^33^	*U*^12^	*U*^13^	*U*^23^
Co1	0.0215 (3)	0.0216 (3)	0.0339 (3)	0.0004 (2)	0.0054 (2)	−0.0016 (2)
Cl1	0.0384 (5)	0.0396 (5)	0.0696 (6)	−0.0099 (3)	0.0126 (4)	−0.0209 (4)
S1	0.0224 (4)	0.0263 (4)	0.0332 (4)	−0.0016 (3)	0.0051 (3)	0.0001 (3)
O1	0.0275 (12)	0.0230 (11)	0.0555 (15)	−0.0012 (10)	0.0120 (11)	−0.0052 (10)
O2	0.0246 (11)	0.0258 (12)	0.0520 (14)	0.0042 (9)	0.0105 (10)	0.0054 (10)
O3	0.0261 (11)	0.0372 (11)	0.0375 (12)	−0.0036 (9)	0.0105 (9)	−0.0032 (9)
O4	0.0326 (11)	0.0247 (10)	0.0413 (12)	−0.0052 (8)	0.0061 (10)	0.0010 (9)
N1	0.0284 (13)	0.0287 (13)	0.0373 (14)	0.0042 (10)	0.0063 (11)	−0.0024 (11)
N2	0.0241 (13)	0.0237 (12)	0.0401 (14)	0.0002 (9)	0.0026 (11)	0.0004 (11)
N3	0.0413 (16)	0.0428 (15)	0.0343 (14)	0.0065 (12)	0.0082 (12)	0.0040 (12)
N4	0.0235 (12)	0.0295 (13)	0.0377 (14)	0.0019 (10)	0.0017 (11)	−0.0023 (11)
N5	0.0261 (13)	0.0296 (13)	0.0399 (15)	−0.0022 (10)	0.0034 (11)	−0.0043 (11)
C1	0.0365 (18)	0.0438 (19)	0.0411 (19)	−0.0041 (14)	0.0075 (15)	0.0011 (15)
C2	0.049 (2)	0.055 (2)	0.041 (2)	0.0012 (17)	0.0155 (17)	0.0091 (17)
C3	0.050 (2)	0.056 (2)	0.0301 (17)	0.0174 (18)	0.0056 (16)	−0.0023 (16)
C4	0.046 (2)	0.047 (2)	0.042 (2)	0.0085 (16)	−0.0042 (17)	−0.0120 (16)
C5	0.0353 (17)	0.0331 (16)	0.0422 (18)	−0.0005 (13)	0.0014 (15)	−0.0027 (14)
C6	0.0198 (14)	0.0253 (14)	0.0334 (16)	−0.0004 (11)	0.0049 (12)	0.0000 (12)
C7	0.0295 (16)	0.0374 (17)	0.0360 (17)	0.0054 (13)	0.0093 (14)	−0.0033 (14)
C8	0.0316 (17)	0.0368 (17)	0.0379 (18)	0.0097 (13)	0.0063 (14)	0.0014 (14)
C9	0.0294 (16)	0.0269 (15)	0.0325 (16)	−0.0039 (12)	0.0046 (13)	−0.0014 (13)
C10	0.0341 (17)	0.0353 (17)	0.0390 (17)	0.0016 (13)	0.0159 (14)	−0.0013 (14)
C11	0.0284 (16)	0.0299 (16)	0.0442 (18)	0.0059 (12)	0.0132 (14)	−0.0003 (14)
C12	0.0245 (14)	0.0260 (14)	0.0318 (16)	0.0021 (11)	0.0053 (12)	0.0017 (12)
C13	0.0270 (15)	0.0292 (16)	0.0394 (17)	−0.0045 (12)	0.0095 (13)	−0.0053 (13)
C14	0.0293 (16)	0.0250 (15)	0.0505 (19)	0.0052 (12)	0.0070 (14)	−0.0020 (14)
C15	0.0211 (15)	0.0292 (15)	0.0463 (18)	0.0015 (11)	0.0025 (13)	0.0002 (14)

### Geometric parameters (Å, °)

**Table:**

Co1—O2^i^	2.028 (2)	C1—C2	1.379 (5)
Co1—O2	2.028 (2)	C1—H1C	0.9500
Co1—O1	2.142 (2)	C2—C3	1.365 (5)
Co1—O1^i^	2.142 (2)	C2—H2C	0.9500
Co1—N1^i^	2.176 (2)	C3—C4	1.374 (5)
Co1—N1	2.176 (2)	C3—H3	0.9500
Cl1—C13	1.737 (3)	C4—C5	1.378 (5)
S1—O4	1.448 (2)	C4—H4	0.9500
S1—O3	1.461 (2)	C5—H5	0.9500
S1—N2	1.577 (2)	C6—C7	1.384 (4)
S1—C6	1.759 (3)	C6—C11	1.387 (4)
O1—H1A	0.81 (4)	C7—C8	1.376 (4)
O1—H1B	0.83 (4)	C7—H7	0.9500
O2—H2A	0.82 (3)	C8—C9	1.392 (4)
O2—H2B	0.82 (4)	C8—H8	0.9500
N1—C1	1.331 (4)	C9—C10	1.398 (4)
N1—C5	1.346 (4)	C10—C11	1.375 (4)
N2—C12	1.368 (3)	C10—H10	0.9500
N3—C9	1.374 (4)	C11—H11	0.9500
N3—H3A	0.8800	C12—C15	1.418 (4)
N3—H3B	0.8800	C13—C14	1.386 (4)
N4—C12	1.347 (4)	C14—C15	1.352 (4)
N4—N5	1.352 (3)	C14—H14	0.9500
N5—C13	1.306 (4)	C15—H15	0.9500
			
O2^i^—Co1—O2	180.0	C1—C2—H2C	120.5
O2^i^—Co1—O1	87.63 (9)	C2—C3—C4	118.8 (3)
O2—Co1—O1	92.37 (9)	C2—C3—H3	120.6
O2^i^—Co1—O1^i^	92.37 (9)	C4—C3—H3	120.6
O2—Co1—O1^i^	87.63 (9)	C3—C4—C5	119.0 (3)
O1—Co1—O1^i^	180.00 (4)	C3—C4—H4	120.5
O2^i^—Co1—N1^i^	88.56 (9)	C5—C4—H4	120.5
O2—Co1—N1^i^	91.44 (9)	N1—C5—C4	123.1 (3)
O1—Co1—N1^i^	89.87 (9)	N1—C5—H5	118.4
O1^i^—Co1—N1^i^	90.13 (9)	C4—C5—H5	118.4
O2^i^—Co1—N1	91.44 (9)	C7—C6—C11	119.4 (3)
O2—Co1—N1	88.56 (9)	C7—C6—S1	120.8 (2)
O1—Co1—N1	90.13 (9)	C11—C6—S1	119.5 (2)
O1^i^—Co1—N1	89.87 (9)	C8—C7—C6	120.1 (3)
N1^i^—Co1—N1	180.000 (1)	C8—C7—H7	120.0
O4—S1—O3	114.83 (12)	C6—C7—H7	120.0
O4—S1—N2	105.51 (12)	C7—C8—C9	121.1 (3)
O3—S1—N2	113.63 (13)	C7—C8—H8	119.5
O4—S1—C6	106.39 (13)	C9—C8—H8	119.5
O3—S1—C6	106.34 (13)	N3—C9—C8	120.5 (3)
N2—S1—C6	109.90 (13)	N3—C9—C10	121.1 (3)
Co1—O1—H1A	120 (3)	C8—C9—C10	118.3 (3)
Co1—O1—H1B	124 (2)	C11—C10—C9	120.5 (3)
H1A—O1—H1B	111 (3)	C11—C10—H10	119.8
Co1—O2—H2A	125 (3)	C9—C10—H10	119.8
Co1—O2—H2B	120 (3)	C10—C11—C6	120.5 (3)
H2A—O2—H2B	111 (4)	C10—C11—H11	119.7
C1—N1—C5	116.5 (3)	C6—C11—H11	119.7
C1—N1—Co1	123.0 (2)	N4—C12—N2	113.2 (2)
C5—N1—Co1	120.4 (2)	N4—C12—C15	120.3 (3)
C12—N2—S1	121.93 (19)	N2—C12—C15	126.5 (3)
C9—N3—H3A	120.0	N5—C13—C14	124.8 (3)
C9—N3—H3B	120.0	N5—C13—Cl1	115.6 (2)
H3A—N3—H3B	120.0	C14—C13—Cl1	119.6 (2)
C12—N4—N5	120.4 (2)	C15—C14—C13	117.0 (3)
C13—N5—N4	118.8 (2)	C15—C14—H14	121.5
N1—C1—C2	123.7 (3)	C13—C14—H14	121.5
N1—C1—H1C	118.2	C14—C15—C12	118.7 (3)
C2—C1—H1C	118.2	C14—C15—H15	120.6
C3—C2—C1	119.0 (3)	C12—C15—H15	120.6
C3—C2—H2C	120.5		
			
O2^i^—Co1—N1—C1	−57.6 (2)	O3—S1—C6—C11	173.7 (2)
O2—Co1—N1—C1	122.4 (2)	N2—S1—C6—C11	−62.9 (3)
O1—Co1—N1—C1	−145.2 (2)	C11—C6—C7—C8	−1.6 (4)
O1^i^—Co1—N1—C1	34.8 (2)	S1—C6—C7—C8	173.1 (2)
O2^i^—Co1—N1—C5	119.1 (2)	C6—C7—C8—C9	−1.0 (5)
O2—Co1—N1—C5	−60.9 (2)	C7—C8—C9—N3	−178.7 (3)
O1—Co1—N1—C5	31.5 (2)	C7—C8—C9—C10	2.8 (5)
O1^i^—Co1—N1—C5	−148.5 (2)	N3—C9—C10—C11	179.5 (3)
O4—S1—N2—C12	173.4 (2)	C8—C9—C10—C11	−2.1 (4)
O3—S1—N2—C12	46.8 (3)	C9—C10—C11—C6	−0.5 (5)
C6—S1—N2—C12	−72.3 (3)	C7—C6—C11—C10	2.3 (4)
C12—N4—N5—C13	−0.2 (4)	S1—C6—C11—C10	−172.5 (2)
C5—N1—C1—C2	−0.3 (5)	N5—N4—C12—N2	178.8 (2)
Co1—N1—C1—C2	176.4 (3)	N5—N4—C12—C15	−1.3 (4)
N1—C1—C2—C3	0.1 (5)	S1—N2—C12—N4	−176.0 (2)
C1—C2—C3—C4	0.0 (5)	S1—N2—C12—C15	4.2 (4)
C2—C3—C4—C5	0.1 (5)	N4—N5—C13—C14	1.2 (5)
C1—N1—C5—C4	0.4 (5)	N4—N5—C13—Cl1	−178.8 (2)
Co1—N1—C5—C4	−176.4 (2)	N5—C13—C14—C15	−0.7 (5)
C3—C4—C5—N1	−0.3 (5)	Cl1—C13—C14—C15	179.3 (2)
O4—S1—C6—C7	−123.9 (2)	C13—C14—C15—C12	−0.9 (5)
O3—S1—C6—C7	−1.0 (3)	N4—C12—C15—C14	1.8 (5)
N2—S1—C6—C7	122.4 (2)	N2—C12—C15—C14	−178.3 (3)
O4—S1—C6—C11	50.8 (3)		

Symmetry codes: (i) −*x*+2, −*y*+1, −*z*+1.

### Hydrogen-bond geometry (Å, °)

**Table:**

*D*—H···*A*	*D*—H	H···*A*	*D*···*A*	*D*—H···*A*
N3—H3A···N5^ii^	0.88	2.52	3.135 (4)	127
N3—H3B···O3^iii^	0.88	2.23	3.001 (3)	147
O1—H1A···O3	0.81 (4)	2.07 (4)	2.874 (3)	174 (4)
O1—H1B···N4^iv^	0.83 (4)	2.01 (4)	2.838 (3)	178 (3)
O2—H2A···O4	0.82 (3)	1.93 (4)	2.726 (3)	165 (4)
O2—H2B···N2^v^	0.82 (4)	1.96 (4)	2.768 (3)	170 (4)
O2—H2B···O4^v^	0.82 (4)	2.62 (4)	3.142 (3)	123 (3)

Symmetry codes: (ii) *x*+1, *y*, *z*+1; (iii) *x*, *y*, *z*+1; (iv) *x*+1, *y*, *z*; (v) −*x*+1, −*y*+1, −*z*+1.
